# Patterns in melanocytic lesions: impact of the geometry on growth and transport inside the epidermis

**DOI:** 10.1098/rsif.2014.0339

**Published:** 2014-08-06

**Authors:** Thibaut Balois, Clément Chatelain, Martine Ben Amar

**Affiliations:** 1 Laboratoire de Physique Statistique, Ecole Normale Supérieure, UPMC Univ Paris 06, Université Paris Diderot, CNRS, 24 rue Lhomond, Paris 75005, France; 2 Faculté de médecine, Institut Universitaire de Cancérologie, Université Pierre et Marie Curie-Paris 6, 91 Boulevard de l'hôpital, Paris 75013, France

**Keywords:** pattern formation, tumour multiphase model, skin cancer morphology, clinical dermatology, geometrical growth and transport

## Abstract

In glabrous skin, nevi and melanomas exhibit pigmented stripes during clinical dermoscopic examination. They find their origin in the basal layer geometry which periodically exhibits ridges, alternatively large (limiting ridges) and thin (intermediate ridges). However, nevus and melanoma lesions differ by the localization of the pigmented stripes along furrows or ridges of the epidermis surface. Here, we propose a biomechanical model of avascular tumour growth which takes into account this specific geometry in the epidermis where both kinds of lesions first appear. Simulations show a periodic distribution of tumour cells inside the lesion, with a global contour stretched out along the ridges. In order to be as close as possible to clinical observations, we also consider the melanin transport by the keratinocytes. Our simulations show that reasonable assumptions on melanocytic cell repartition in the ridges favour the limiting ridges of the basal compared with the intermediate ones in agreement with nevus observations but not really with melanomas. It raises the question of cell aggregation and repartition of melanocytic cells in acral melanomas and requires further biological studies of these cells *in situ*.

## 1. Introduction

Melanomas are the most deadly skin cancer, being responsible for 75% of the mortality due to skin cancer according to the American Cancer Society.^1^ Unlike cancers affecting other organs, these tumours are directly observable since the primary tumour appears as a pigmented lesion at the surface of the skin. Early detection is therefore made possible by simple skin examination, eventually performed by the patient himself. When a melanoma is detected at an early stage, it can be treated by simple excision and the 10 year survival rate is higher than 99%. However, the survival rate drops to less than 50% when it penetrates deeply into the dermis. In the last decades, many efforts have been made to improve the methods of differential diagnosis in order to classify malignant and benign melanocytic lesions based on morphological criteria. Empirical studies from collections of clinical cases have led to the identification of shapes and microstructures, especially thanks to more and more precise tools like dermoscopy [1,2]. But the mechanisms generating these structures as the morphological differences between malignant and benign tumours remain largely unknown. We recently proposed physical mechanisms controlling the contour regularity of melanocytic tumours [3–5] and explaining the apparition of microstructures such as pigmented dots and globules [6]. However, the models that have been developed until now suppose that the various layers of the skin are horizontal with the avascular growth occurring in a thin epidermis with a simple flat geometry. In certain regions of the body, the skin has a more pronounced geometry, often associated with specific microstructures, such as dermal papillae. We discuss here how the skin geometry can influence the pattern formation in melanocytic tumours. The effect of the geometry on growth has already been demonstrated for morphogenesis experimentally [7] and theoretically [8].

**Figure 1.**
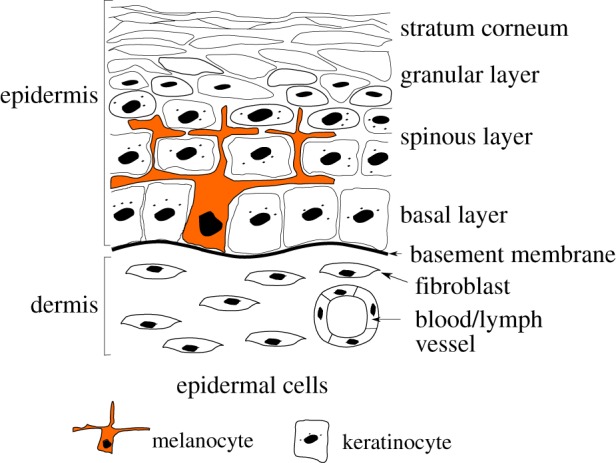
Schematic of the two outmost layers of the skin, the dermis and the epidermis.

Human skin can be divided into three layers, epidermis, dermis and hypodermis. The epidermis is the superficial layer of the skin and is mainly composed of keratinocytes and to a less extent of melanocytes (figure 1). Keratinocytes proliferate in a basal monolayer attached to the basement membrane forming the dermal–epidermal junction, and migrate towards the skin surface during their differentiation. The stratum corneum is the outermost part of the epidermis and is made of fully differentiated keratinocytes and of non-living corneocytes, in a lipid-rich matrix regulating skin permeability. In healthy tissue, each melanocyte remains connected to neighbour keratinocytes and to the basement membrane. Its main role consists of producing the pigment of the skin called melanin. Melanin is enclosed in vesicles and is then transported by neighbour keratinocytes via endocytosis and exocytosis. Melanocytic lesions such as nevi and melanoma originate from a dysregulation of melanocytes leading to the invasion of the surrounding tissue. The structure of human skin varies significantly depending on the location on the body. We can define two main types of skin: non-glabrous and glabrous skin. Non-glabrous skin is characterized by the presence of hair follicles and a thin epidermis (100 µm thick typically) almost planar except near the hair follicles. Hairless skin, which is found on the palms and soles, is instead characterized by a thick epidermis (1 mm thick typically), including a dense and thick stratum corneum. At the skin surface, dermatoglyphs are formed by a periodic alternation of ridges and furrows [9]. At the dermal–epidermal junction, an epidermal ridge lies under each surface furrow and each surface ridge: the limiting ridges (*crista profunda limitans*) and intermediate ridges (*crista profunda intermedia*), respectively. Intermediate ridges are narrower than the limiting ridges [10] and host the eccrine sweat glands (*acrosyringium*) that appear by dermoscopy as white dots at the centre of each surface ridge [11]. This complex structure plays an important role in the perception of touch and induces a strong mechanical coupling between the dermis and epidermis in palms and soles allowing the skin to support high mechanical stresses [12]. Among non-white populations, glabrous skin is the most common location for melanoma. For instance, in Japanese population 50% of detected melanomas are found in these locations [13]. Clinical research has shown that nevi and melanomas developing on glabrous skin have specific shapes, certainly influenced by the geometry of the skin in these locations, and requiring appropriate diagnostic criteria.

On glabrous skin, nevi and melanomas *in situ* are generally associated with parallel pigmented stripes. For nevi, these stripes are usually located along the furrows of the skin surface, parallel furrow patterns (40–45% of acral nevi [14]), unlike melanomas which are associated with similar pigmented parallel patterns but located along the ridges of the skin surface, parallel ridge patterns (83% of melanoma *in situ*) [14–17]. Saida *et al.* [14] reported a sensitivity of 86% and a specificity of 99% for the early diagnosis of acral melanomas using an algorithm based on these parallel patterns. These shapes suggest a strong influence of the geometry of the epidermis on the distribution of melanocytes and melanin in agreement with clinical observations [10,18]. For instance, using electron microscopy, Nagashima & Tsuchida [10] have observed the geometry of the dermal–epidermal junction at different sites of the feet and have shown that on each site, the pigmentation pattern of nevi follows the structure of epidermal ridges.

A tissue section of acral nevus performed perpendicularly to skin fingerprints frequently shows the presence of melanin columns extending from the limiting ridge of the dermal–epidermal junction towards the skin surface [19]. A preferential proliferation of nevus cells in the limiting ridges has been proposed to explain the presence of these columns. However, recent investigations by Palleschi *et al.* [20] and by Saida *et al.* [17] show that this explanation is not sufficient. In some acral nevi, these authors show that aggregates of nevus cells are present in both limiting and intermediate ridges, but columns of melanin are found only above limiting ridges. Inhibition of melanin synthesis in intermediate ridges could explain this phenomenon.

In this paper, we aim to model such clinical observations. As dermascopes detect the melanin pigment and not the melanocytes, we consider the proliferation of tumour cells on a distorted basement membrane and also the transport of the originated melanin in the same geometry. For this purpose, we develop two mathematical models. Both models take into account the existence of epidermal ridges. These models provide a geometrical explanation for the apparition of parallel patterns in melanocytic lesions of glabrous skin and for the localization of melanin columns above limiting ridges. In §2, we revisit the avascular growth model for melanomas [3–6] taking into account the geometry of the epidermis. In §3, we propose a new model for melanin transport and melanin column distribution inside the epidermis. In §4, we discuss the physical results which correctly describe the nevi stripes, but not completely the pigmented stripes of acral melanoma. In appendix A, we give a more detailed description of the methods used in this study, a list of symbols is given in table 1 and a list of parameter values is given in table 2.

**Table 1.** Nomenclature.

*n* nutrient concentration in three dimensions
*ε* thickness of the thin layer of proliferation
*N* nutrient concentration in two dimensions
*ϕ*_c_ melanocytic cells concentration
*ϕ*_h_ healthy phase concentration (keratinocytes)
*κ* nutrient consumption of keratinocytes (*ϕ*_e_)
*v*_c_ velocity of *ϕ*_c_
*Γ* mass exchange rate between *ϕ*_c_ and *ϕ*_h_
*Ψ* free-energy between the melanocytic cells
∑ derivative of the free-energy *Ψ*
*f*(*ϕ*_c_) volumic term of ∑
*𝒦* mobility constant of the melanocytic phase *ϕ*_c_
*g_ij_* metric tensor of the surface *S*_B_
*g* = det(*g_ij_*) basement membrane
*H* mean radius of curvature *S*_B_
*S*_B_ proliferation surface corresponding to the basal layer
SC epidermis outer surface (stratum corneum)
*k* permeability of the keratinocytes phase
*μ* viscosity of the keratinocytes phase
*K* = *k*/*μ* Darcy constant
*p* hydrostatic pressure
*v* local rate of migration of keratinocytes
*V* migration rate from the basal layer (boundary condition)
*c* melanin concentration
*c*_0_ melanin concentration in the basal layer (boundary condition)
*D* diffusion coefficient of melanin
*δ*_m_ degradation rate of melanin
*A* amplitude of the basal layer oscillation
*Z*_nest_ height of the melanocytic cells nest
*p*_0_ homeostatic pressure

**Table 2.** List of symbols used in §2 and estimation of the model parameters from experimental data on healthy and diseased skin.

*ϕ*_e_ cell volume fraction at mechanical equilibrium	0.6–0.9	[39]
*M* interphase friction	963–11 571	[40,41]
*χ* interstitial fluid pressure in healthy skin	133 Pa	[39]
*χ* interstitial fluid pressure in skin carcinoma	1330 Pa	[39]
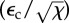 melanoma cell size	6*–*20 µm	[42]
*γ*_c_ melanoma cell proliferation rate	0.2 d*^−^*^1^	[43]
*δ*_c_ threshold for cell death rate due to anoxia	0.1−0.33	[44]
*D_n_* oxygen lateral diffusion coefficient	39.7 mm^2^ d*^−^*^1^	[45]
*D_n_* oxygen perpendicular diffusion coefficient	18.5*–*26.6 mm^2^ d*^−^*^1^	[46]
*D_n_* sugar diffusion coefficient	4.4*–*6.4 mm^2^ d*^−^*^1^	[47]
*δ_n_* oxygen consumption rate of the skin	1190*–*3310 d*^−^*^1^	[46]
*D_z_* perpendicular diffusion constant	10^4^ d*^−^*^1^	[5]
*n*_s_, *N*_1_, *N*_2_ oxygen partial pressure in the skin	3320*–*10 400 Pa	[46]
*l*_n_ nutrient penetration length	0.04*–*0.18 mm	
*D* potential strength in equation (2.9)	1.7*–*1725	
*ε*_c_ surface tension in equation (2.9)	0.02*–*0.5	
*δ* death rate of *ϕ*_c_ in equation (2.9)	0.1*–*0.33	
*β*_1_ perpendicular diffusion in equation (2.8)	0.1*–*8.4	
*κ* nutrient consumption of *ϕ*_h_ in equation (2.8)	0.1*–*10	
*n*_1_, *n*_2_ external nutrient concentration in equation (2.8)	0.25*–*4.	

## 2. Model of melanoma cell proliferation on curved surfaces

In order to understand the clinical observations of melanocytic lesions on glabrous skin, we first focus on cell proliferation and tumour growth. Several approaches have been developed to model tumour growth [21–23]. We develop a continuous model based on the theory of mixtures [24,25]. Even though there have been studies of diffusion on a curved surface [26] and proliferation of curved epithelium [27], no cancer models take into account the curvature of the epithelium. For simplicity sake, this mixture model takes into account only two phases [4,6]: a cancerous proliferative phase with a concentration *ϕ*_c_ and a second healthy phase containing the interstitial fluid, the dead cells and the keratinocytes with a concentration *ϕ*_h_ = 1 − *ϕ*_c_. The concentration represents the percentage of each kind of cell at a given point inside the lesion, but an average is achieved on a scale larger than the cell scale. Both phases need nutrients to ensure cell functions. In this section, we present briefly the chosen geometry of glabrous skin and we detail the governing equations induced by the curvature effects of the limiting basement membrane. These equations can be treated only numerically and the simulations will be compared to clinical observations.

### 2.1. Geometry and governing equations

**Figure 2.**
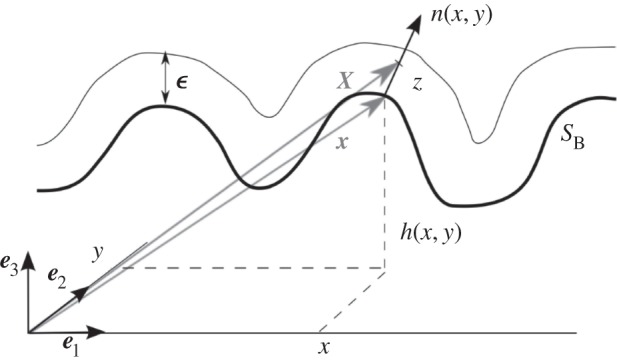
Monge representation of the surface *S*_B_ and representation of the thin layer of thickness *ε*. A point on the surface *S*_B_ is represented by ***x*** = *x**e***_1_ + *y**e***_2_ + *h*(*x*, *y*)***e***_3_, and the normal vector to the surface *S*_B_ at ***x***(*x*, *y*) is written as ***n***(*x*, *y*). The thin layer is represented by the set of points ***X*** = ***x*** + *z**n*** with 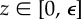.

We consider the growth of a thin layer with thickness *ε* on a curved surface *S*_B_. *S*_B_ represents the basement membrane, and the layer is constituted of the proliferative zone containing melanocytes, so basically the spinous and basal layer of the epidermis. Its order of magnitude is of a few cells size, so few times 6 μm. Therefore, we can consider the layer as two dimensional and the fraction of cancerous phase *ϕ*_c_(*x*, *y*) at the point of coordinates (*x*, *y*), once averaged does not depend on *z*. In the following, we use the Monge representation of the surface *S*_B_, which means that the vertical coordinate of one point of the surface is *z*_B_ = *h*(*x*, *y*) (figure 2). The classical equations of tumour growth [3–5] are modified by this geometry. The induced modifications are rather technical requiring the tools of differential geometry. It turns out that it has never been treated before in the literature of tumour modelling perhaps because of its specificity to skin cancers. A complete description of curvature effects on differential mathematical operators is given in appendix A.

Inside the thin layer, the small nutrient molecules of concentration *n* diffuse and are consumed by the cells. The timescale for diffusion being much shorter than the uptake time, the diffusion is treated at equilibrium, and we derive the following three-dimensional equation:2.1
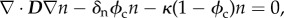
where ***D*** is the diffusion matrix, with coefficients *D* = *D_||_* when the diffusion occurs along the surface *S*_B_, and *D* = *D*_⊥_ when the diffusion occurs perpendicularly. *δ*_n_ (resp. *κ*) is the nutrient consumption rate by the cancerous phase (resp. by the healthy phase). The thickness *ε* being small, an averaged description of all quantities is enough for our purpose restoring a two-dimensional description. It is why we integrate this equation along the *z*-axis giving2.2
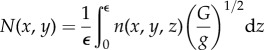
and2.3
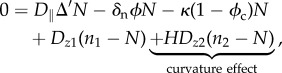
where *N*(*x*, *y*) is the surface concentration at (*x*, *y*); *G*, *g* and *H* are metric tensors given in appendix A. Δ′ is the Laplace–Beltrami operator (see equation (A 15)); *n*_1_ and *n*_2_ are typical values of the nutrient concentration outside the thin layer (table 2). The first term of equation (2.3) represents the diffusion along the curved surface, whereas the second and third terms represent the orthogonal diffusion. Note that the third term due to the curvature will change sign along the surface. Although small, it is positive for ridges and increases the orthogonal flux and the local nutrient concentration as well. The two last terms represent nutrient consumption. Compared to previous works, the novelty of such equations concerns the real treatment of the basal geometry. Note that the averaging process allows recovery of a two-dimensional tractable equation, at least for simulations, without loss of pertinent information.

**Figure 3.**
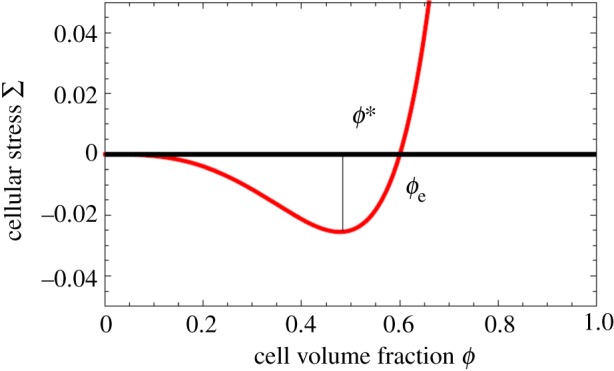
∑, derivative of the free-energy as a function of concentration, *f*(*ϕ*_c_) representing the attraction between melanocytic cells. *ϕ*_e_ is the concentration at equilibrium with the surrounding tissue.

As we have assumed the same mass density for the two phases *ϕ*_c_ and *ϕ*_h_, the mass exchanges between them are the same, and we can focus on the mass balance equation for *ϕ*_c_2.4
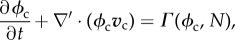
where ***v***_c_ is the velocity of the cancerous phase, and *Γ*(*ϕ*_c_, *N*) is the mass exchange rate. Using a variationnal principle, ***v***_c_ is related to the derivative of the free-energy between cancer cells ∑(*ϕ*_c_) [5,6]2.5
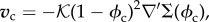
with 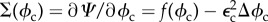, *Ψ*(*ϕ*_c_) being the free-energy between melanocytic cells. As expected for a cell–cell interaction, the volumic contribution *f*(*ϕ*_c_) in ∑ is weak at low concentration, attractive at intermediate concentration and becomes repulsive. Thus, it has a simple representation reminiscent of the Lennard–Jones potential (figure 3). A surface term 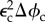 is added to ∑(*ϕ*_c_) penalizing large concentration gradients.

For the mass exchange rate *Γ*(*ϕ*_c_, *N*), a simple linear law is assumed: *Γ*(*ϕ*_c_, *N*) = *γ*_c_*ϕ*_c_(*N*/*n*_s_ − *δ*) with death and proliferation rate, *δ* and *γ*_c_, being constant. Therefore, the final two-dimensional equation for the cell concentration is given by2.6
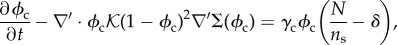
with the Laplace–Beltrami operator (appendix A) noted with a superscript ′ given by2.7
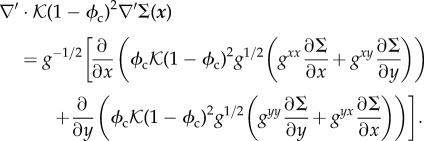
We rewrite equations (2.3) and (2.6) with dimensionless quantities: 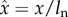 and 
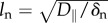
 being the nutrient penetration length, 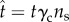 and 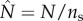. Finally, dropping hats and adapting all coefficients accordingly, we get the two coupled master equations to solve2.8
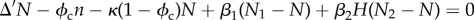
and2.9
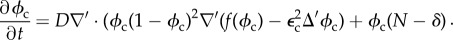
The first mathematical symbol of equation (2.8) represents the lateral diffusion on a curved surface (equation (A 15)). The second (resp. the third) term accounts for the nutrient consumption of the melanocytic cells (resp. the healthy cells, mostly keratinocytes). The fourth term is the main term of the transversal diffusion, and the fifth term is the curvature contribution to the transversal diffusion. In equation (2.9), we recover the terms of the mass balance equation (equation (2.4)), the velocity in the convection term is calculated on a curved surface (equation (2.7)) and the third term expresses the cell proliferation and death. Only numerical simulations will allow recovery of some features of clinical observations. Note that table 2, which gives biomechanical quantities measured in the literature, makes the model quantitative as well.

### 2.2. Numerical results

Numerical simulations of equations (2.8) and (2.9) were performed in a two-dimensional *N* × *N* lattice, with *ϕ*_c_ ≡ 0 and *n* ≡ *n*_0_ at the lattice border. The initial conditions are chosen to follow parabolic distribution for the concentration with addition of an initial white noise. For the simulations, we choose *f* in ∑ as2.10
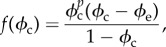
where 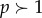, in order to represent the form of figure 3. Here, we fix *p* = 2 and *ϕ*_e_ = 0.6. The simulations are executed in the following steps:

— calculate the value of ∑ on the grid and
— implement the cell density (resp. the nutrient) value using a forward time centred space scheme to represent equation (2.9) (resp. equation (2.8)). A time dependence with a different timescale has been introduced in equation (2.8) to ensure that growth is slower than diffusion.

**Figure 4.**
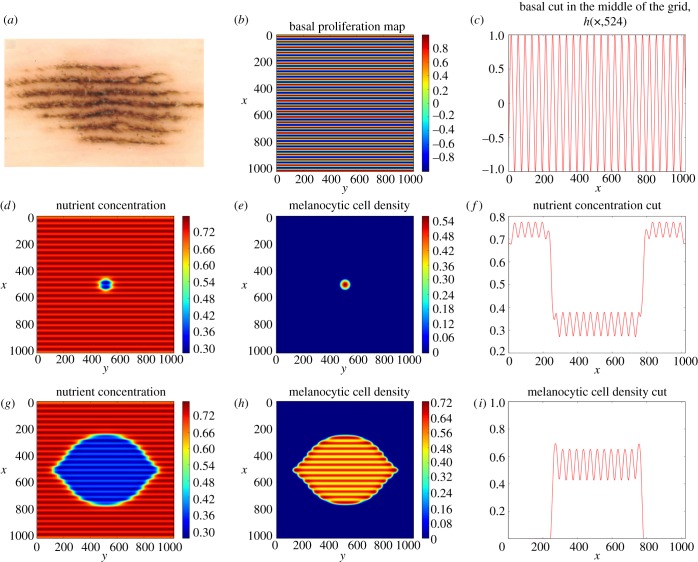
(*a*) Dermoscopy picture of an acral nevi taken from [14]. We estimate the lesion on the surface to be roughly 4.8 mm wide and 10 mm long. Pictures (*b*–*i*) come from a simulation made on a 1024 × 1024 lattice. The chosen parameters are: *δ* = 0.333333, *D* = 2.0, *β*_1_ = 0.3, *β*_2_ = 0.03, *κ* = 0.1, *n*_2_ = 1.0, *ε*_c_ = 0.4, *h*_0_ = 1.0, the time-step *δt* = 0.00005 and the size step *δ_x_* = 0.1. (*b*) Pictures of the basal layer *h*(*x*, *y*), which is the basal proliferation map. (*e*,*h*) Pictures of the cell distribution at time *t* = 0 and 200. (*d*,*g*) Pictures of the nutrient distribution at time *t* = 5 and *t* = 200. The *x-* and *y-*axes give the grid position. The wavelength of the basal perturbation is 4*l_n_*, approximately 0.4 mm. The size of the computed lesion on the basal represented in (*h*) is approximately 4 mm wide and 7 mm long. Pictures (*c*,*f*,*i*) are one-dimensional representation of, respectively, the basal proliferation map, the nutrient and the cell density, in the middle of the grid (the 524th column).

The program was implemented on a graphic processor unit, a GTX-580 NVIDIA graphic card, using the CUDA parallel programming language, in order to reduce computational time (appendix A.3.1). The value of parameters is chosen inside the range of biological values reported in table 2. The form of the basement membrane *S*_B_ is taken regular and sinusoidal (figure 4*b*) with *h*(*x*, *y*) = *h*_0_cos(*kx*) = *h*_0_cos(*k'i*), where *i* is the line position on the grid.

An example of the simulations is given in figure 4. The simulated patterns follow the ridges of the basal pattern and are elongated along its direction although initial conditions are circular. The pattern of figure 4*h* resembles the pattern of the acral nevus shown by dermoscopy in figure 4*a*. As expected, the nutrient distribution plays an important role in this matter, as growth is driven by nutrient diffusion in the avascular phase and only *N* is modified by the concavity of the surface *S*_B_. We focused on a small range of parameters in order to avoid the effect of a phase separation process [6], corresponding to a small value of *D* and an important value for *ε*_c_. We chose a value of the height of the basal perturbation *h*_0_ = 1.0 *l*_n_ (roughly 100 µm which is around the *in vivo* value). The parameters *δ*, *β*_1_ and *κ* regulate the concentration of the melanocytic cells inside the lesion [28].

In this section, we have proposed a model and simulations exhibiting the importance of the geometry of the wavy basement membrane. Considering the small thickness of the epidermis, a two-dimensional description remains valid when the geometry is accurately taken into account. The fact that the equations are written in two-dimensional space dimensions is of utmost importance for simulations. Indeed, the basal geometry modifies mostly the nutrient repartition and by consequence the cell proliferation, with a higher cell density in the epidermal ridges. The cancerous cell repartition follows the basement geometry as shown in our numerical study and also clinically. However, one needs to remember that the signal of the dermascope used in clinical patient examination is not sensitive to the tumour cell concentration but to the melanin repartition in the whole depth of the epidermis. This melanin repartition also results from the basement geometry, where it is initially produced. It is why we also consider the transport of melanin from the basement membrane up to the upper layers of the epidermis.

## 3. Melanin transport in the epidermis

Several models both experimental and mathematical have been developed to describe the transport of molecules through the skin. To our knowledge, nothing has been done for the transport of melanin. Our model is based on the transport of the melanin vesicles convected by the displacement of keratinocytes. It means that we need to consider the dynamics of keratinocytes first.

### 3.1. Model of keratinocyte migration and melanin transport in the epidermis

**Figure 5.**
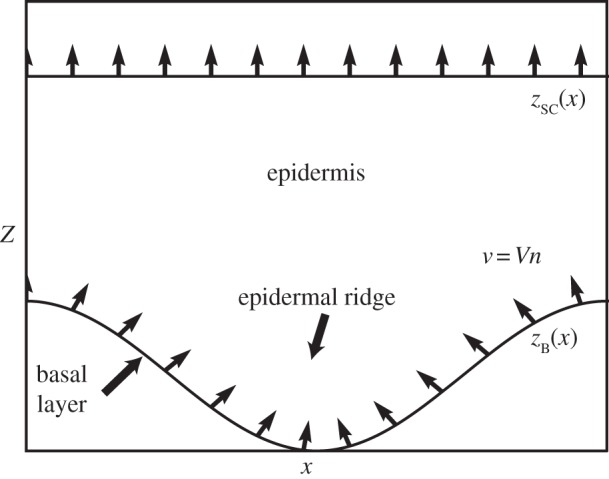
Schematic geometry of the epidermis and of our melanin transport model. The dermo-epidermal junction *S*_B_ supports the basal layer where keratinocytes proliferate and where melanocytes synthesize melanin. On glabrous skin, the structure of dermo-epidermal junction consists of an oscillating height *z*_B_(*x*). Keratinocytes migrate along the normal to the basal layer (apical migration) with a migration rate *V* towards the skin surface SC where they are eliminated by desquamification. Arrows represent migration rate of keratinocytes *v*.

We need to represent the migration of keratinocytes from the basal layer *S*_B_, where they proliferate, towards the skin surface SC, where they are removed by desquamation. The surfaces *S*_B_ and SC are described by the equations *z* = *z*_B_(*x*) and *z* = *z*_SC_(*x*) (respectively, taking an *x-*axis parallel to the skin surface and a vertical axis *z*, figure 5). Of course no physical model exists for the displacement of the cells inside the epidermis so we take constitutive equations commonly used in transport of viscous fluids. We discuss here two types of constitutive equations known as the Darcy and Stokes flow. The Darcy description allows analytical resolution, but is more restrictive. Whereas the Stokes flow can only be analysed through numerical solutions, but its applications are broader.

Assuming at first the migration inside a porous medium, the steady flow of keratinocytes is given by the Darcy equation3.1
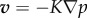
and3.2
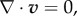
with *p* the hydrostatic pressure, ***v*** the local rate of migration of keratinocytes proportional to the pressure gradient *∇p*, *K* = *k*/*μ*, *k* the permeability *μ* the viscosity of the tissue.

To demonstrate the generality of the observed mechanisms, we have considered in a second step a migration process described by a Stokes flow, which is more adapted to highly viscous and incompressible flow, and is given by the equations3.3
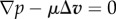
and3.4
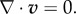


From the basal layer *S*_B_, keratinocytes migrate along the normal to this surface (apical migration) imposing the boundary conditions for the two different flow descriptions3.5
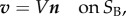
where ***n*** is the normal to the surface of *S*_B_, and *V* the migration rate from the basal layer.

The concentration of melanin in the basal layer *c* = *c*_0_ depends on the rate of synthesis of the melanin by melanocytes in this layer. It diffuses actively between the cells [29] and is convected with the migration of keratinocytes described by the velocity field ***v***. Experimental studies suggest that melanin degradation occurs at a constant rate *δ*_m_ and is almost complete when reaching the skin surface SC [30,31]. The melanin concentration can therefore be described by a convection–diffusion equation3.6
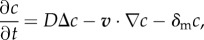
with the boundary conditions3.7
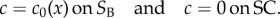
A typical value for the three terms of equation (3.6) can be estimated from the literature. Thingnes *et al.* [32] give an estimate of the degradation rate of melanin *δ*_m_ ∼ 10*^−^*^2^ h*^−^*^1^. Dover [33] reports the typical migration time of a keratinocyte from the basal layer to the skin surface *T*_T_ = 154 − 641 h which leads to an estimate of the convection term *|**v*** · ***∇**|* ∼ *V*/*H* ∼ 5 × 10*^−^*^3^ h*^−^*^1^, with *H* ∼ 1 mm the thickness of the epidermis and *V* ∼ 37 − 155 µm · d*^−^*^1^ the typical migration rate of keratinocytes. The transfer of melanin between keratinocytes has been investigated by Singh *et al.* [29] who have observed a transfer in 24 h through filopodia of typical length 20 µm. We deduce from these experiments an estimate of the diffusion coefficient *D* ∼ 16 µm^2^ · h*^−^*^1^ and of the diffusion term in equation (3.6) *|D*Δ*|* ∼ *D*/*H*^2^ ∼ 1.6 × 10*^−^*^5^ · h*^−^*^1^. The comparison of these experimental values shows that the diffusion term in equation (3.6) can be neglected and this equation can be rewritten simply as3.8
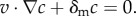


### 3.2. Keratinocyte migration and melanin transport

#### 3.2.1. Darcy flow

**Figure 6.**
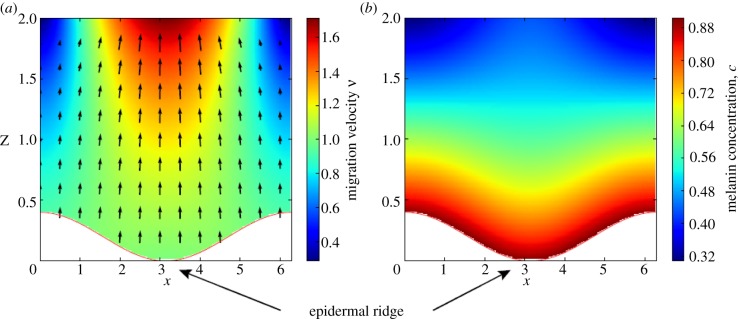
Effect of geometry on the migration of keratinocytes (*a*) and on the concentration of melanin *c* in the epidermis (*b*). The basal layer *S*_B_, separating the dermis and the epidermis has an undulated geometry given by the equation *z* = *z*_B_(*x*) = *A*(1+cos(*kx*)). Keratinocytes leave this layer with a constant velocity *V* along the normal to the layer. Representing the migration of keratinocytes by a Darcy flow, the stationary velocity *v* of these cells is given analytically to first order in *A* by equations (3.14) and (3.15) and are shown in (*a*). The concentration of melanin is *c*_0_ = 1.0 in the basal layer *S*_B_, where pigments are produced by melanocytes. Taking into account advection and degradation of melanin, the steady-state concentration is given analytically to first order in *A* by the equation (3.16) and is shown in figure (*b*). Approximate solutions to order *A* are shown for *A* = 0.2, *k* = 1.0, *V* = 1.0 and *δ*_m_ = 0.5, and all quantities are dimensionless.

We first consider a keratinocyte migration represented by a Darcy flow and a simple geometry3.9
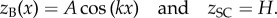
This type of flow is well suited to the description of a viscous flow in a porous medium and has the advantage that it can be treated analytically [34]. The velocity field *v* can be represented by a velocity potential *ϕ* satisfying the following equations:3.10
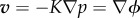
and3.11
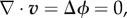
with the boundary conditions3.12
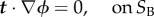
and3.13
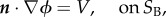
with ***t*** the tangent to surface *S*_B_. Assuming a uniform migration rate *V* in the basal layer, the problem can be solved analytically close to the surface *S*_B_ where we find the keratinocyte velocity3.14
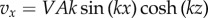
and3.15
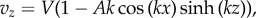
at the first order in *A* (figure 6). Assuming a uniform melanin concentration *c*_0_ in *S*_B_, the steady distribution of melanin advected by the keratinocyte migration is given at the order *A*3.16
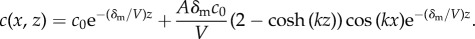
As diffusion has been neglected in the transport equation, the concentration of melanin depends only on the concentration in the basal layer *c*_0_ and the speed evolution *v* along the current lines. Assuming that melanin is rapidly degraded (*δ*_m_/*v* > *k* and 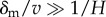), the apparent skin pigmentation at first order *A* can be obtained by integrating this concentration along *z* between *z*_B_ and +*∞*3.17
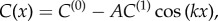
with *C*^(0)^ the average pigmentation and *C*^(1)^ > 0 the amplitude of the corrections due to geometrical constraints on melanin transport. Equation (3.17) shows that *C*(*x*) is in phase opposition with *z*_B_(*x*), indicating that even in the case where the production of melanin is uniform in the basal layer a parallel pigmented pattern appears with a stronger pigmentation above the epidermal ridges. This result shows that the patterns observed in melanocytic lesions of glabrous skin can be explained by geometric constraints on melanin transport. The keratinocyte migration is faster above the epidermal ridges due to the local pressure (figure 6), as a consequence the vertical advection and dispersion of melanin is therefore increased.

To demonstrate the generality of the mechanism for appearance of the pigmented parallel pattern illustrated above, we consider now a keratinocyte migration described by a Stokes flow.

#### 3.2.2. Stokes flow

**Figure 7.**
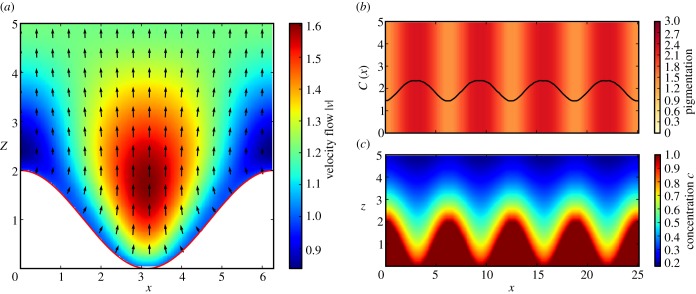
(*a*) Velocity field *v* for the migration of keratinocytes in the epidermis, assuming a stationary Stokes flow with a constant velocity *V* along the normal of the basal layer. The dermal–epidermal junction has an undulated geometry given by the equation *z*_B_(*x*) = *A*(1 + cos(*kx*)). As for the Darcy flow (figure 6), the keratinocyte migration is faster above the epidermal ridges than above the furrows. (*c*) Steady-state concentration of melanin *c*(*x*, *y*). The pigment is produced in the basal layer, where *c* = *c*_0_ and advected by the migration of keratinocytes as described by equation (3.18). Owing to the faster migration above epidermal ridges, melanin distribution is wider above these regions. (*b*) Resulting pigmentation observed on the surface of the skin and defined by 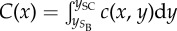. Even in the case of a uniform production of pigment in the basal layer, geometric constraints on melanin transport leads to the apparition of a parallel pattern with stronger pigmentation above epidermal ridges. Numerical solutions are shown for *A* = *k* = *δ*_m_ = *V* = 1.0 and all quantities are dimensionless.

We assume a similar form for *S*_B_ and the same boundary as previously ***v*** = *V**n*** on *S*_B_, i.e. the same apical migration constant, and ***v*** = (*v_x_*, *v_z_*) = (0, *V*′) on SC, where *V’* is chosen such that the incompressibility constraint can be satisfied. A numerical simulation of discretized equations (3.3) and (3.4) has been performed in Python using a relaxation method (appendix A.3.2). The results are shown in figure 7. As in the case of Darcy flow, the keratinocyte velocity is more important above the epidermal ridges than above the furrows. This difference of velocity causes a faster advection of melanin above these regions and the emergence of parallel patterns in skin pigmentation, even for a uniform production of melanin in the basal layer. In the case of the thick skin of hairless areas, 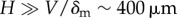, the concentration of intact melanin in the upper layers of the epidermis is negligible, the result presented here is independent of the form chosen for the interface SC and the results are qualitatively the same for other values of the parameters. The numerical studies confirm that the emergence of parallel grooves can be explained only by the geometric constraints imposed on the transport of melanin as shown in figure 7.

### 3.3. Localization of melanin column and stress inhibition of keratinocyte proliferation

**Figure 8.**
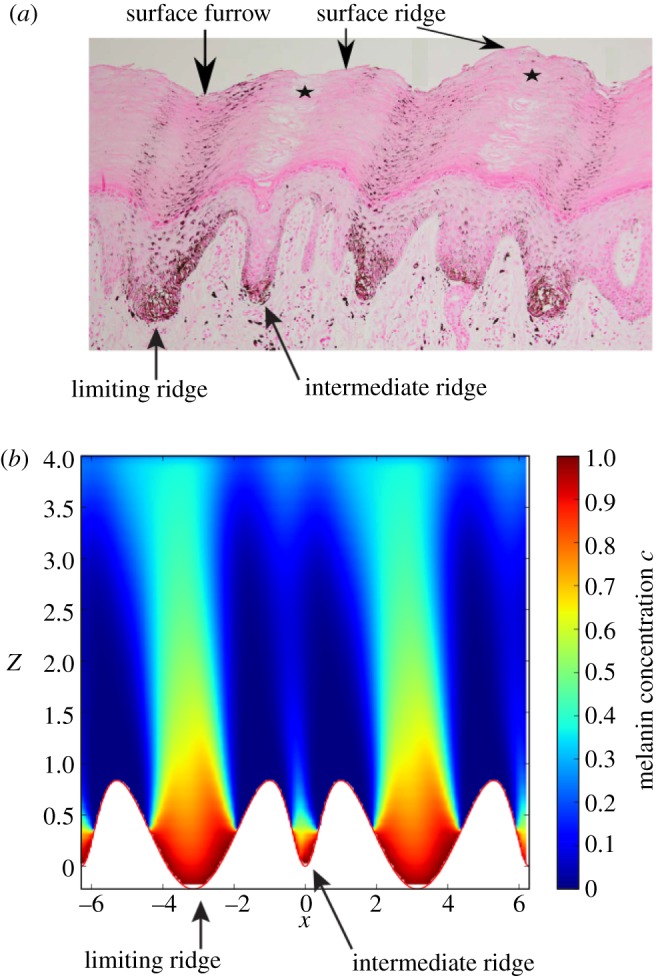
(*a*) Histopathological features of an acral nevi with Fontana-Masson staining showing the distribution of melanin granules in the epidermis. Clusters of melanocytic cells are present in the epidermal ridges. Columns of melanin appear only above limiting ridges, resulting in the parallel furrow pattern typical of nevi. Figure adapted from [17]. (*b*) Numerical simulations of the distribution of melanin given by equations (3.3), (3.4) and (3.8). Melanin is produced in the epidermal ridges (*z* < *Z*_nest_), the concentration of melanin on *S*_B_ is *c* = *c*_0_ in these areas and *c* = 0 outside. The pigments are advected by the migration of keratinocytes and degraded at a constant rate *δ*_c_. Proliferation of keratinocytes in the basal layer *S*_B_ is regulated by the local pressure to maintain an homeostatic pressure *p*_0_. Keratinocytes migrate along the normal to the basal layer. This migration is described by a Stokes flow with a viscosity *μ*. The migration velocity *v* is lower above the intermediate ridges (narrower than the limiting ridges) due to the non-slip condition and the constant pressure imposed at the boundary *S*_B_. The equation of the dermal–epidermal junction is *z*_B_(*x*) = *A*(1 + cos(*x*) − 2/(1 + 8sin(*x*/2)^2^)) and the model parameters are *Z*_nest_ = *A*/3.0, *A* = 1.0, *δ*_m_ = 0.5, *μ* = 1.0 and *p*_0_ = 1.0. All quantities are dimensionless.

The dermal–epidermal junction of the hairless skin consists of a periodic alternance of narrow ridges (intermediate ridges) and wide ridges (limiting ridges). Even if nevus cells tend to form aggregates in these two types of ridges, only the melanin produced in the limiting ridges is transported to the upper layers of the epidermis eventually making melanin columns (figure 8). We adapt here the geometry and the boundary conditions of our model to provide an explanation for this phenomenon.

To take into account the accumulation of melanocytes in epidermal ridges as suggested by the study of §2, the following boundary conditions on *S*_B_ is chosen for the concentration of melanin:3.18
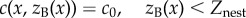
and 3.19
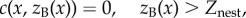
with *Z*_nest_ a constant describing the vertical extension of the melanocyte nest.

Many experiments have highlighted the important regulatory effect of mechanical stress on cell proliferation (see [35] for instance). We take now into account the inhibition of keratinocyte proliferation by the mechanical pressure assuming a homeostatic pressure *p*_0_ in the basal layer. A local pressure *p* > *p*_0_ leads to a decrease in proliferation and migration velocity, and a pressure *p* < *p*_0_ stimulates proliferation and increases the rate of migration [36]. Boundary conditions on *S*_B_ for the Stokes flow are therefore modified as3.20
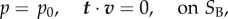
and the boundary conditions on SC are unchanged.

The numerical solution for melanin concentration obtained in a realistic epidermis geometry is shown in figure 8. In the narrow ridges, fast keratinocyte migration induces a high pressure on the basal layer, due to the non-slip assumption on this layer, leading to an inhibition of the proliferation and a decrease of the migration rate. In a steady state, migration rates are therefore much lower in the narrow intermediate ridges, than in the wide limiting ridges. As discussed previously, the vertical dispersion of melanin increases with the migration rate of keratinocytes, which explains why our model predicts the apparition of melanin columns only above limiting ridges.

## 4. Discussion

In most cases, melanocytic lesions in hairless skin have a notable structure and exhibit pigmented parallel stripes, usually attributed to the specific geometry of the skin in these areas. However, there are differences in the position of the stripe network between nevi and melanomas. In nevi, it is believed that the pigmented stripes coincide with the surface furrows while it would be the contrary for melanomas, giving perhaps a possible diagnosis. The mechanisms explaining the appearance of these structures, however, remain largely mysterious [20]. Here, we present two biomechanical models which explain physically such structures: one is based on tumour growth modelling, the other on melanin repartition inside the epidermis, both of them being strongly influenced by the basal geometry.

The first model considers the avascular growth of the lesion due to cancer cell proliferation in layers strongly distorted by the basal geometry. Focusing on a periodic undulated basal at the origin of fingerprints on soles and palms, we establish first a model able to accurately take into account such structure, then we perform simulations. It turns out that the nutrient concentration which decreases in the lesion due to consumption by the proliferative cells is more important in the valleys compared with the crests, increasing the proliferation in the ridges but also the activity of the tumour cells like perhaps melanin production. This effect is locally dependent on the curvature, so increases in narrow ridges compared with wide ridges.

Our second model focuses more on the transport of melanin in such glabrous areas of the skin. For that we take into account the specific transport of this pigment synthesized in the basal layer, advected to the upper layers of the epidermis by the apical migration of keratinocytes and degraded at a constant rate. The model has been solved analytically and numerically in the case of uniform melanin production on an undulated basal layer. It shows that the geometry of the basal surface influences the migration rate of keratinocytes which is predicted to be faster above epidermal ridges and leads to the apparition of a parallel pattern in the apparent skin pigmentation, with stronger pigmentation above the epidermal ridges. This corresponds to the clinical observation for nevi and shows that this pigmentation pattern can be explained only by the constraints imposed by the geometry of the epidermis on the keratinocyte migration. However, we note that histopathological studies show that melanocytes tend to aggregate and the assumption of uniform melanin synthesis is therefore probably not true as suggested by our first model. Parallel patterns along ridges are due to the combination of aggregation of melanocytic cells and melanin transport as suggested by our two models.

In nevi, nevus cells can be found in both intermediate and limiting ridges [17,20], but it was noted that only the melanin synthesized in limiting ridges is transported to the upper layers of the epidermis to form eventually a melanin column forming parallel furrow pattern. Using our model of melanin transport, we have proposed an explanation of this phenomenon based on the inhibition of keratinocyte proliferation by mechanical stress. The epidermis being treated as a viscous medium for migration, keratinocyte migration in narrow ridges lead therefore to significant constraints in the basal layer, attached to the dermal–epidermal junction, which inhibits cell proliferation and reduces the cellular migration rate as well as the advection of melanin. This explanation has been validated numerically and the results correspond to the melanin distribution observed in histopathology. As for melanomas, melanin transport should obey the same rules as in our model and as for nevi.

Therefore, the position of parallel ridges for melanoma cannot be explained by physical reasons alone. Only biological facts can justify them and perhaps be included in our study in the future. However, they are still missing to our knowledge. For instance, one questionable hypothesis would be the boundary condition on melanin distribution in equations (3.18) and (3.19). We can argue that the increase of nutrients in intermediate ridges would result in a higher cell activity and melanin production, but it is not sure that this physical argument is enough to justify the dichotomy between melanomas and nevi. However, more research on the biology of melanocytes *in situ* is necessary. Indeed, the melanocytic precursor cells originate from the dermis [37,38]. A possibility would be a preference of melanoma stem cells to migrate in intermediate ridges. These models enlighten the dominating mechanisms behind the parallel patterns, but they need to be improved by more biological information on the location and activities of the different melanocytic cells in order to be able to accurately predict the behaviour of a lesion and fully explain the significance of the current diagnostic tools.

## Endnote

1 American Cancer Society. Cancer Facts and Figures 2013. http://www.cancer.org/acs/groups/content/@epidemiologysurveilance/documents/document/acspc-036845.pdf. (Accessed 31 January 2013.)

## Appendix A

### A.1. Differential geometry of the basement membrane

Using the same notations as in §2.1, we describe the metric allowing us to differentiate on a curved surface. Following Ogawa [26], we write (*G_ij_*) the three-dimensional metric tensor at first order in *ε*

Therefore, we can write (*G_ij_*) as

A1

and

A2

with *g_ij_* is the metric tensor of the surface *S*_B_ as

A3

and *κ_ij_* is the second fundamental tensor,

A4

where . At the first order in *ε*, we find

A5

with *H* the mean radius of curvature of *S*_B_,

A6

In our case, we only consider a dependence on *x*. So the expression of (*g_ij_*), *g* and *H* is as follows:

A7

A8

and

A9

### A.2. Nutrient diffusion

Here, we detail the integration along the *z*-axis that allows a two-dimensional description of nutrients diffusion. The amount of nutrient contained in a section of surface *S′* of the thin layer is written as

A10

Therefore, we use the average nutrient concentration in two-dimensional *N*(*x*, *y*) at (*x*, *y*)

A11

We choose a nutrient concentration depending weakly on the *z*-axis but with sharp variations

A12

thus we have

A13

In order to find a two-dimensional description of equation (2.1), we multiply it by (*G*/*g*)^1/2^ and integrate it over *z* between 0 and *ε*. The first term gives us

A14

where (*G^ij^*) = (*G_ij_*)*^−^*^1^. The horizontal diffusion is equal to Δ′*n*, where Δ′ is the standard Laplace–Beltrami operator.

A15

with (*g^ij^*) = (*g_ij_*)*^−^*^1^

A16

The vertical diffusion is integrated, and the flux at the border is supposed proportional to the difference between the concentration inside the layer *N*(*x*, *y*) and the concentration outside *n*_d_ and *n*_e_

The integration of the consumption term allows us to recover equation (2.8). As in our example, we have *h*(*x*, *y*) = *h*(*x*), the derivation process is simplified and equations (2.8) and (2.9) become

A17

and

A18

### A.3. Numerical scheme

#### A.3.1. Scheme of melanocytic cells growth

The numerical simulation are done by discretizing equations (2.8) and (2.9) on a two-dimensional lattice. In the case of the simple form of the surface chosen (*h*(*x*, *y*) = *h*(*x*)), we consider the nutrient field , the cells field , the interaction field  and the derivation fields   and  (the mean radius of *S*_B_), where *k* is the time index, and *i* and *j* are the grid position. The first step of the simulation is to calculate the value of , we use the following scheme for the Laplace–Beltrami operator:

A19

In order to express equation (2.8), we use the same scheme to express Δ′ and we introduce a time dependence with a different timescale to ensure that growth is slower than diffusion

A20

we chose . We use the same approach to implement  using Δ*t* instead of . These schemes were written in CUDA and the results were drawn using Python.

#### A.3.2. Scheme of the Stokes flow model

We use a relaxation method to solve the stationary Stokes equation on the domain *Ω* with the following algorithm written in Python:

— initialization of *p_i_*_,*j*_, *v_x_*_,*i*,*j*_ and *v_y_*_,*i*,*j*_ respecting the boundary conditions;
— *p_i_*_,*j*_ is calculated to satisfy the Laplace equation Δ*p* = 0 using the relaxation method;
— *v_x_*_,*i*,*j*_ and *v_y_*_,*i*,*j*_ are determined by solving Δ*v_x_* = *∂_x_p*/*μ* and Δ*v_y_* = *∂_y_p*/*μ* using the relaxation method and
— calculation of the incompressibility error *dp* = *∇* · *v* by finite differences. If the error does not satisfy the convergence criterium, i.e. , we implement *p*,  and return to the third step. Otherwise the resolution is over.

### A.4. Biological data and symbols

An estimation of the parameters has been gathered in table 2. We also give an estimation of the dimensionless parameters. These quantities are used in our simulations. A list of symbols of §2 and §3 can be found in table 1.
